# Pilot Evaluation of *S*-(3-[^18^F]Fluoropropyl)-d-Homocysteine and *O*-(2-[^18^F]Fluoroethyl)-d-Tyrosine as Bacteria-Specific Radiotracers for PET Imaging of Infection

**DOI:** 10.1007/s11307-024-01929-7

**Published:** 2024-06-28

**Authors:** Helen M. Betts, Jeni C. Luckett, Philip J. Hill

**Affiliations:** 1 Department of Nuclear Medicine, Nottingham University Hospitals NHS Trust, Queen’s Medical Centre, Nottingham, NG7 2UH UK; 2 School of Medicine, University of Nottingham, Queen’s Medical Centre, Nottingham, NG7 2UH UK; 3 School of Life Sciences, University of Nottingham, Biodiscovery Institute, University Park, Nottingham, NG7 2RD UK; 4 School of Biosciences, University of Nottingham, Sutton Bonington Campus, Sutton Bonington, LE17 5RD UK

**Keywords:** PET, Fluorine-18, Bacterial infection, *S. aureus*, *P. aeruginosa*, d-amino acids

## Abstract

**Purpose:**

There is currently no ideal radiotracer for imaging bacterial infections. Radiolabelled d-amino acids are promising candidates because they are actively incorporated into the peptidoglycan of the bacterial cell wall, a structural feature which is absent in human cells. This work describes fluorine-18 labelled analogues of d-tyrosine and d-methionine, *O*-(2-[^18^F]fluoroethyl)-d-tyrosine (d-[^18^F]FET) and *S*-(3-[^18^F]fluoropropyl)-d-homocysteine (d-[^18^F]FPHCys), and their pilot evaluation studies as potential radiotracers for imaging bacterial infection.

**Procedures:**

d-[^18^F]FET and d-[^18^F]FPHCys were prepared in classical fluorination-deprotection reactions, and their uptake in *Staphylococcus aureus* and *Pseudomonas aeruginosa* was evaluated over 2 h. Heat killed bacteria were used as controls. A clinically-relevant foreign body model of *S. aureus* infection was established in Balb/c mice, as well as a sterile foreign body to mimic inflammation. The *ex vivo* biodistribution of d-[^18^F]FPHCys in the infected and inflamed mice was evaluated after 1 h, by dissection and gamma counting. The uptake was compared to that of [^18^F]FDG.

**Results:**

*In vitro* uptake of both d-[^18^F]FET and d-[^18^F]FPHCys was specific to live bacteria. Uptake was higher in *S. aureus* than in *P. aeruginosa* for both radiotracers, and of the two, higher for d-[^18^F]FPHCys than d-[^18^F]FET. Blocking experiments with non-radioactive d-[^19^F]FPHCys confirmed specificity of uptake. *In vivo*, d-[^18^F]FPHCys had greater accumulation in *S. aureus* infection compared with sterile inflammation, which was statistically significant. As anticipated, [^18^F]FDG showed no significant difference in uptake between infection and inflammation.

**Conclusions:**

d-[^18^F]FPHCys uptake was higher in infected tissues than inflammation, and represents a fluorine-18 labelled d-AA with potential to detect a *S. aureus* reference strain (Xen29) *in vivo*. Additional studies are needed to evaluate uptake of this radiotracer in clinical isolates.

**Supplementary Information:**

The online version contains supplementary material available at 10.1007/s11307-024-01929-7.

## Introduction

Bacterial infection and antimicrobial resistance are recognised as global threats to human health [1, 2]. The aging population coupled with surgical advances means that increasing numbers of patients are undergoing elective surgery—such as joint replacements, vascular grafts and cardiac implants—and there has been a concomitant increase in the number of hospital-acquired infections [3, 4]. Approximately 800,000 hospital-acquired surgical site infections (SSI) were reported in an EU/EAA survey of 2011–12, leading to 16,000 deaths [5]. The impact of infection for patients ranges from the need to have repeat surgery, leading to immobilisation and weeks’-long hospital stays, to (particularly in the case of vascular graft infections) death [5, 6]. To ensure that patients receive optimal treatment when infection is suspected, and to responsibly manage antibiotic use, rapid, accurate diagnosis of infection is critical.

When patients experience complications post-surgery, conclusive diagnosis of infection is not straightforward. Biopsy sampling from a surgical site is invasive, subject to sampling errors and potential contamination, is dependent on the site being accessible, and fails to account for any heterogeneity [7]. Non-invasive imaging techniques MRI and CT provide limited anatomical clues to the presence of an infection, but these physical changes are often slow to manifest leading to delay in diagnosis. Furthermore, the structural abnormalities observed by these methods can be a result of sterile inflammatory processes (such as prosthetic joint loosening) [8, 9]. There are difficulties in distinguishing active infection from sterile inflammation when using existing clinical radiotracers for positron emission tomography (PET) such as 2-deoxy-2-[^18^F]fluoro-D-glucose ([^18^F]FDG) [10], or single photon emission computed tomography (SPECT), such as [^111^In]In-oxine or [^99m^Tc]Tc-HMPAO labelled white blood cells [11]. Although these radiotracers can be used to detect certain infections with high sensitivity, their uptake is primarily determined by the immune response to infection, and is not specific to the bacteria. Use of these radiotracers is particularly challenging in vulnerable patients who are immune compromised, or have cancer [7, 10–12].

PET imaging using a bacteria-specific radiotracer would provide an ideal technique for diagnosing bacterial infection, as well as have potential to monitor the efficacy of antibiotic treatments. Recent developments towards this goal include probes based on sugar alcohols [13, 14], polysaccharides [15, 16], muramic acid [17] and antibiotics [18], amongst others [19]. First steps in patient imaging have been made with the research radiotracers *p*-aminobenzoic acid ([^11^C]PABA), [^11^C]trimethoprim and [^18^F]fluorodeoxysorbitol ([^18^F]FDS). PABA is a substrate of bacterial folate synthesis and its radiolabelled derivative accumulates in both Gram positive and Gram negative bacteria in all growth phases [20–22]. First in human studies, however, showed rapid metabolism in plasma [23]. [^11^C]Trimethoprim is a radiolabelled antibiotic that has recently shown promising results in patients with confirmed bacterial infections, including those with antibiotic-resistant strains. One of its limiting features for routine use, however, is its short lived carbon-11 radiolabel (t_1/2_ = 20 min) [18]. [^18^F]FDS is selectively metabolised in Enterobacteriaceae such as *E. coli* and can identify these select pathogens by PET in patients, but it is not able to detect Gram positive bacteria such as *S. aureus* [24, 25]. Clinical results of bacteria-specific fluorine-18 radiotracers that can accumulate in Gram positive bacteria are yet to be reported.

**Scheme 1:**
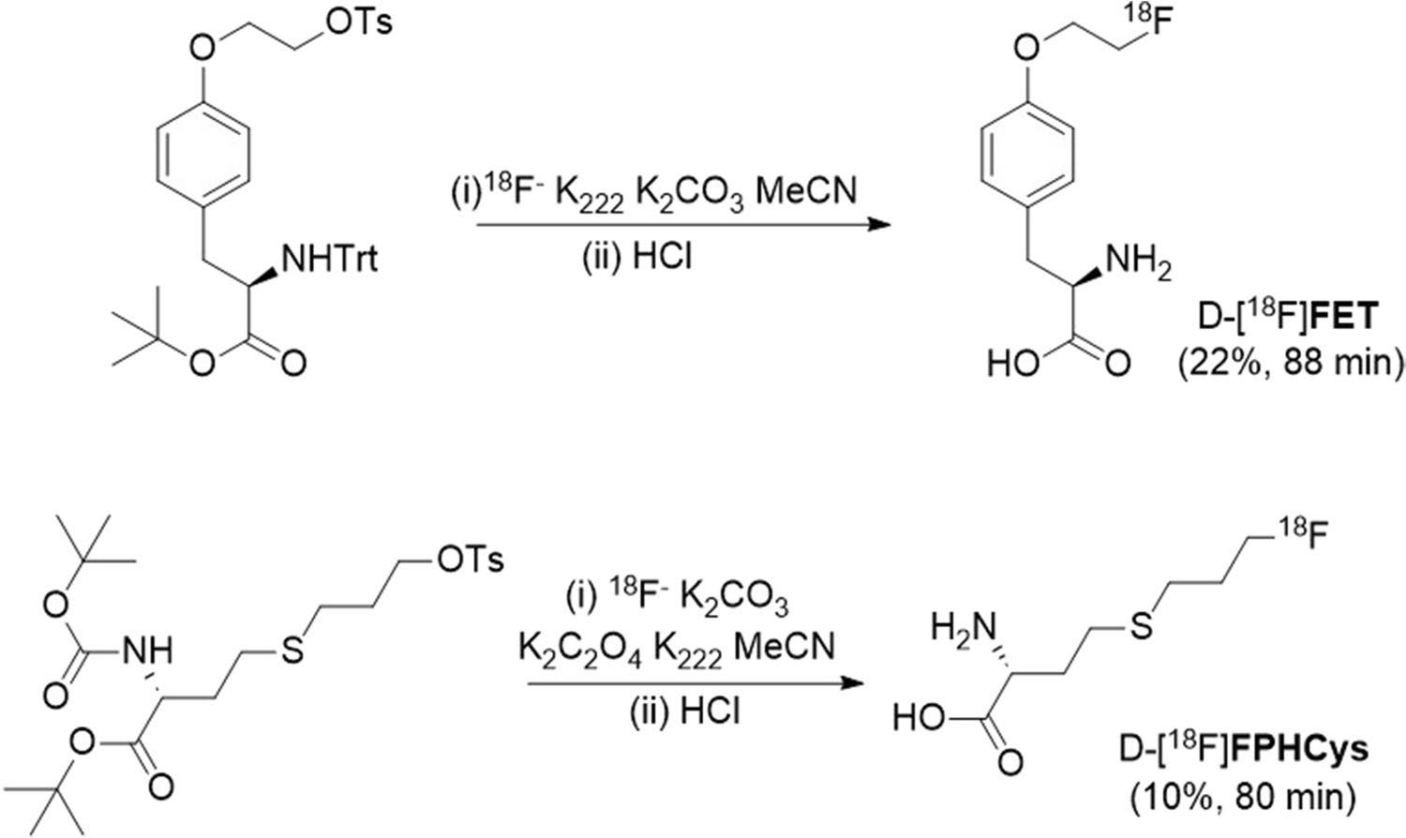
Radiosynthesis of d-[^18^F]FET and d-[^18^F]FPHCys. Radioactivity yields of purified product are given, not corrected for decay.

Recently, d-enantiomers of amino acids (AAs) have emerged as molecules of interest for bacteria-specific imaging. d-AAs are an essential component of bacterial peptidoglycan cell walls, a structural feature which is absent in human cells, whereas d-AAs have limited uses in humans. Notably, d-serine is synthesised by racemase in the human brain and is as co-agonist of the NMDA receptor [26]. The mirror-image l-AAs are, however, prevalent as they are required by human protein synthesis mechanisms. The carbon-11 labelled d-AAs d-methionine (d-[^11^C]Met) [27, 28], d-alanine (d-[^11^C]Ala) [29] and d-glutamine (d-[^11^C]-Gln) [30] have been evaluated as potential bacteria-specific radiotracers, targeting the bacterial cell wall. These radiotracers show specific accumulation in various metabolically active bacteria, but all are limited by the short-lived nature of carbon-11. A fluorine-18 (t_1/2_ = 110 min) labelled d-AA would provide a practical alternative that could be used in hospitals without a local cyclotron. Recently, 3,3,3-[^18^F]trifluoromethyl-d-alanine (d-[^18^F]CF_3_-Ala) was reported with this goal in mind [31]. d-[^18^F]CF_3_-Ala showed highest accumulation in Gram negative bacteria, especially *E. Coli*. Herein, we present results of two fluorine-18 labelled d-AAs as potential bacteria-specific radiotracers; *O*-(2-[^18^F]fluoroethyl)-d-tyrosine (d-[^18^F]FET) and *S*-(3-[^18^F]fluoropropyl)-d-homocysteine, (d-[^18^F]FPHCys) (Scheme 1).

## Materials and Methods

### Chemistry and Radiochemistry

The non-radioactive reference materials d-[^19^F]FET and d-[^19^F]FPHCys, and their radiolabelling precursors were prepared using modified literature procedures for l-enantiomers (Scheme 1) [32, 33]. d-[^18^F]FET and d-[^18^F]FPHCys were radiolabelled using classical nucleophilic substitution-deprotection reactions. Full experimental details and characterisation data are presented in the Electronic Supplementary Material (ESM).

### *In VitroBacteria* Uptake and Blocking Studies

*S. aureus* (Xen29) or *P. aeruginosa* (PA01, Lausanne) were grown to mid-log phase in Ham’s F12 buffer for SILAC (ThermoFisher). Cells were collected by centrifugation and re-suspended in 10% glycerol/phosphate buffered saline (PBS). Aliquots (20 µL, 1 × 10^7^ CFU) were stored at -80 °C until the experiment, then re-suspended in F12 buffer (20 µL). Prior to use, purified d-[^18^F]FPHCys or d-[^18^F]FET (80 µL, mean 32 kBq, range 25–48 kBq) was diluted (1:9 v/v) with F12 buffer, and added to the thawed aliquot. The final concentration of EtOH was < 0.8%. The mixtures were incubated at 37 °C and at 30, 60, 90 and 120 min, centrifuged (12,000 rpm, 5 min), supernatant separated, and washed with PBS (2 × 100 µL). Counts associated with the pellet and washings were recorded. Heat-killed bacteria (90 °C, 30 min, verified by lack of growth on CFU analysis) were used as controls [27]. Blocking studies were performed for d-[^18^F]FPHCys at 120 min, using the radiotracer (70 µL), bacteria in F12 (20 µL) and non-radioactive d-[^19^F]FPHCys (10 µL, dissolved in F12) to give final concentrations of 0.01–5 mM.

### Animal Models

All applicable institutional and national guidelines for the care and use of animals were followed. All animal experiments were approved by the University of Nottingham Animal Welfare and Ethical Review Board and performed under project licence PP5768261 and personal licences granted by the UK Home Office. ARRIVE guidelines were followed. The procedures were classed as moderate in severity.

Subcutaneously implanted Cytodex beads (Cytodex-1 microcarrier beads, particle size 60–87 µm, Sigma) were used as a model foreign body. The beads were hydrated in PBS, then autoclaved. Female Balb/c mice (mean 21.6 g, range 19–24 g) were prepared with subcutaneous injection of the beads on the flank, either co-injected with *S. aureus* Xen29 (1 × 10^5^ CFU), or alone for sterile inflammation. Mice were kept in IVC cages with access to food and water ad libitum, and were weighed and monitored daily. The infection was established for 4 days prior to the biodistribution study. The *S. aureus* group was imaged daily using an IVIS Spectrum In vivo imaging system (Perkin Elmer), and immediately prior to the biodistribution study. Mice were anaesthetised under isoflurane (4% induction; 1.5% maintenance) during image capture, for 30 s acquisition with small (4) binning and open filter settings. Note that *S. aureus* Xen29 contains the *Photorhabdus luminescens* LuxABCDE operon as a single chromosome insertion, thus the bioluminescence is not lost over time due to bacterial replication.

### *Ex Vivo* Biodistribution Study

For [^18^F]FDG, the biodistribution study was performed in mice with inflammation (n = 6) and *S. aureus* infection (n = 6). For d-[^18^F]FPHCys the study was performed in mice with inflammation (n = 4) and *S. aureus* infection (n = 8). The radiotracer was injected via tail vein (mean 1.9 MBq, range 0.8–3.0 MBq), and after 1 h, animals were sacrificed by sodium pentobarbital and organs of interest collected in pre-weighed vials. Samples were analysed using an automated gamma counter (Hidex). Data is expressed as percent injected dose per gram of tissue (%ID/g), decay corrected to injection time. The injected dose (in cpm) was calculated from a calibration curve that related the measured reading on the dose calibrator (syringe pre- and post-injection, in MBq) to the gamma counter (in cpm). Figures were prepared using Graphpad Prism, and show the mean with standard error (SEM) bars.

### Immunohistochemistry

Samples of sites of infection and inflammation were fixed in 10% formalin-saline solution, and processed for paraffin embedding. Sections (8 µm) were taken and fixed to microscope slides. Sections were processed and stained with haematoxylin and eosin according to standard protocols [34], and visualised using a Hamamatsu NanoZoom slide scanner. Immunohistochemistry was performed on parallel sections, which were rehydrated and assessed for *S. aureus* colonisation by epitope retrieval using trypsin (10 µg/mL) at 37 °C for 10 min. Tissues were washed with PBS and pre-blocked at 37 °C using bovine serum (5% *v/v*) for 1 h, then incubated with primary rabbit antibody to *S. aureus* (BioRad 0300–0084, diluted 1:500) for 2 h at 37 °C. After washing with PBS (3 x), tissue sections were incubated with a secondary anti-rabbit Alexa 555. After washing in PBS (3 x), the sections were incubated in DAPI (300 nm) for 10 min. The sections were again washed in PBS (3 x) and mounted with Fluoromount (Sigma Aldrich). Images were acquired using a Zeiss confocal CD7 imager.

### Statistical Analysis

Statistical analysis was performed using Microsoft Excel. Significance was determined by the two unpaired variables t-test, assuming equal variance. The validity of this assumption was checked using Levene’s test.

## Results

### Radiochemistry

d-[^18^F]FET and d-[^18^F]FPHCys were prepared from their respective tosylate precursors by classic nucleophilic ^18^F-fluorination-deprotection reactions (Scheme 1). By using biocompatible HPLC eluents (EtOH-aqueous sodium phosphate) the purified radiotracers could be used directly in the *in vitro* and *in vivo* studies by dilution in F12 medium or PBS without further reformulation. Stability tests in EtOH-sodium phosphate formulation by HPLC indicated no decomposition for at least 6 h.

### *In Vitro* Bacterial Uptake and Blocking Studies

**Fig. 1:**
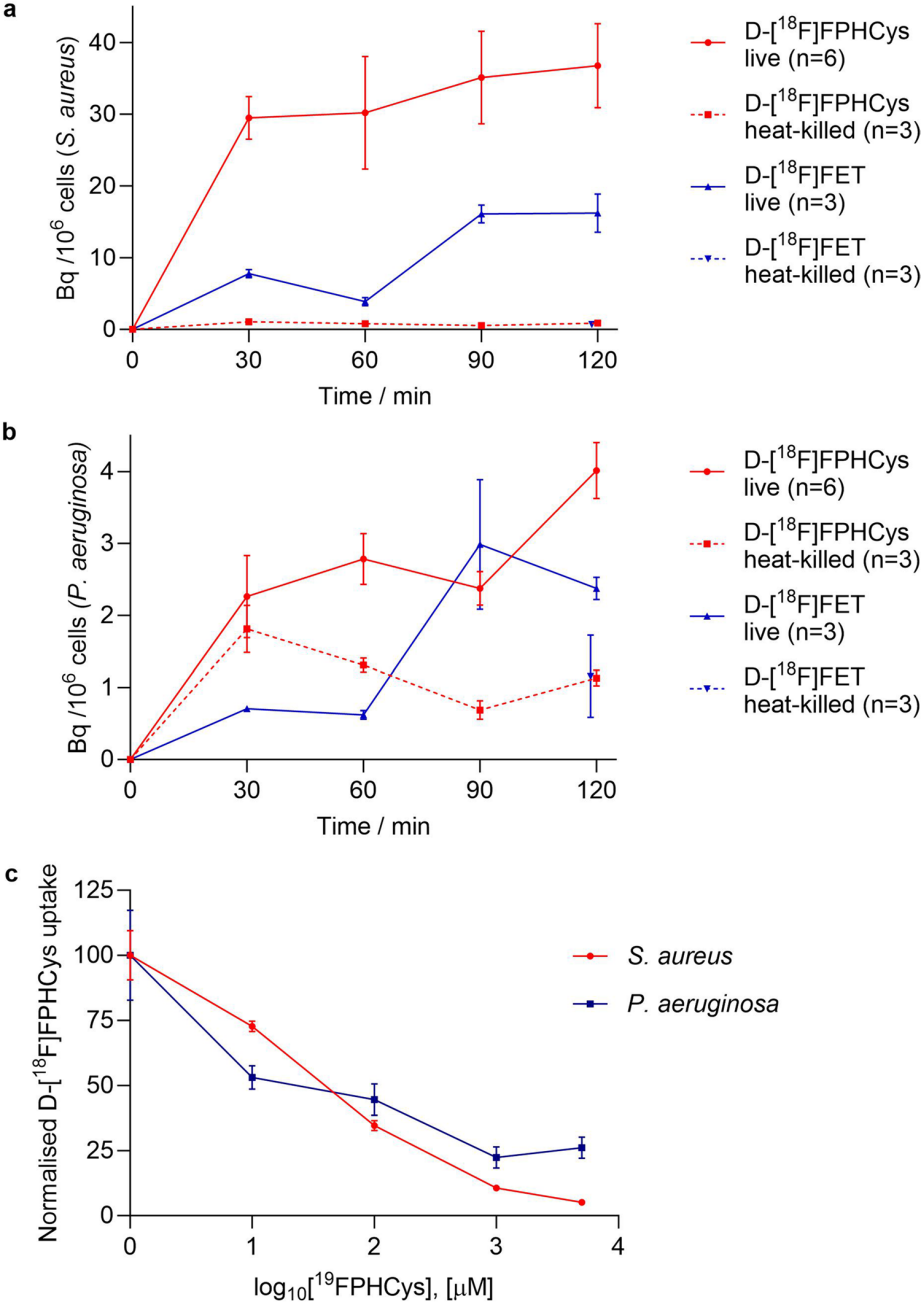
Uptake of d-[^18^F]FET and d-[^18^F]FPHCys in (**a**) *S. aureus* (**b**) in *P. aeruginosa*. NB. d-[^18^F]FET uptake in heat-killed bacteria was performed only at the final time point (2 h). This data point has been slightly offset from 120 min for clarity. Where SEM markers are not visible, the marker falls within the data point. (**c**) Blocking study of d-[^18^F]FPHCys uptake in *S. aureus* and *P. aeruginosa*, where non-radiolabelled d-FPHCys was applied. Graph shows mean with error bars of SEM.

d-[^18^F]FPHCys showed increasing uptake in live *S. aureus* over 2 h (Fig. 1a). At 2 h, the mean uptake reached 36 Bq/10^6^ cells, compared with < 1 Bq/10^6^ cells in heat-killed bacteria at the same time point. d-[^18^F]FPHCys uptake in *P. aeruginosa* was lower (Fig. 1b), reaching 4 Bq/10^6^ cells at 2 h, and 1 Bq/10^6^ cells in heat-killed *P. aeruginosa*. The difference in mean uptake between live and heat-killed bacteria at 2 h was significant in both *S. aureus* and *P. aeruginosa* (*p* = 0.004 and *p* = 0.002 respectively). Data expressed as percentage of applied radioactivity associated with the cells are presented in S5, in the ESM.

d-[^18^F]FET showed similarly increasing uptake over 2 h in *S. aureus*, up to 16 Bq/10^6^ cells in live bacteria compared with < 1 Bq/10^6^ cells in heat-killed *S. aureus* (Fig. 1a). This difference in mean was statistically significant (*p* = 0.004). d-[^18^F]FET uptake in *P. aeruginosa* was lower than in *S. aureus*, reaching 2 Bq/10^6^ cells in live, and 1 Bq/10^6^ cells in heat-killed bacteria, however this was not significant (*p* = 0.11) (Fig. 1b).

Blocking experiments were performed on d-[^18^F]FPHCys uptake to establish the specificity of the incorporation, by addition of d-FPHCys (Fig. 1c). In both *S. aureus* and *P. aeruginosa*, the uptake of d-[^18^F]FPHCys was blocked in a concentration dependent manner.

### Animal Models

**Fig. 2:**
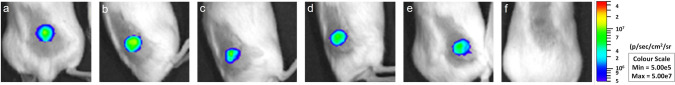
Representative bioluminescence images of mouse with Cytodex beads implanted. (**a-e)** Same mouse with *S. aureus* infection, images on days 0, 1, 2, 3, and 4. (**f**) Mouse with Cytodex bead implant (inflammation) only.

Representative images of the *S. aureus* bioluminescence in a single animal are shown in Figs. 2a-e. Figure 2f shows a mouse implanted with sterile Cytodex beads, confirming no light output in the absence of *S. aureus*.

### *Ex Vivo* Biodistribution Studies

**Fig. 3:**
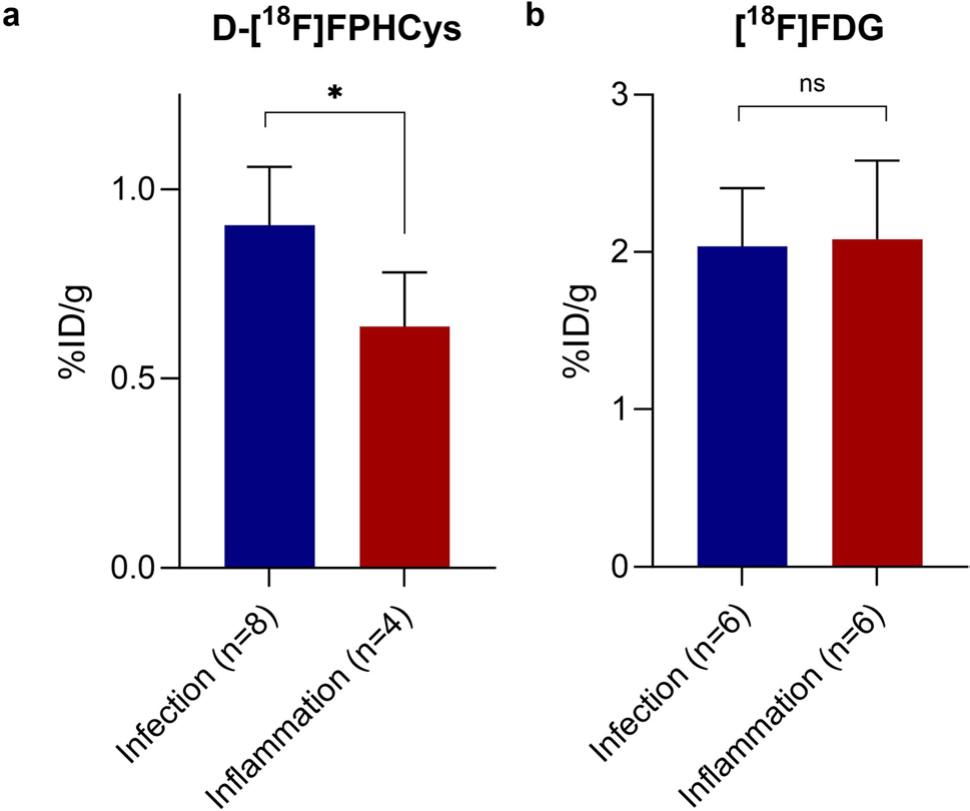
Radiotracer uptake in mouse model of *S. aureus* infection site compared with a sterile inflammation site. (**a**) d-[^18^F]FPHCys (**b**) [^18^F]FDG.

d-[^18^F]FPHCys showed higher mean uptake in the *S. aureus* infection site compared with the inflammation group (*p* = 0.016, Fig. 3a). As expected from the non-specific radiotracer [^18^F]FDG, there was no evidence of a difference in the means in the infection and inflammation sites (Fig. 3b) [10].

**Fig. 4:**
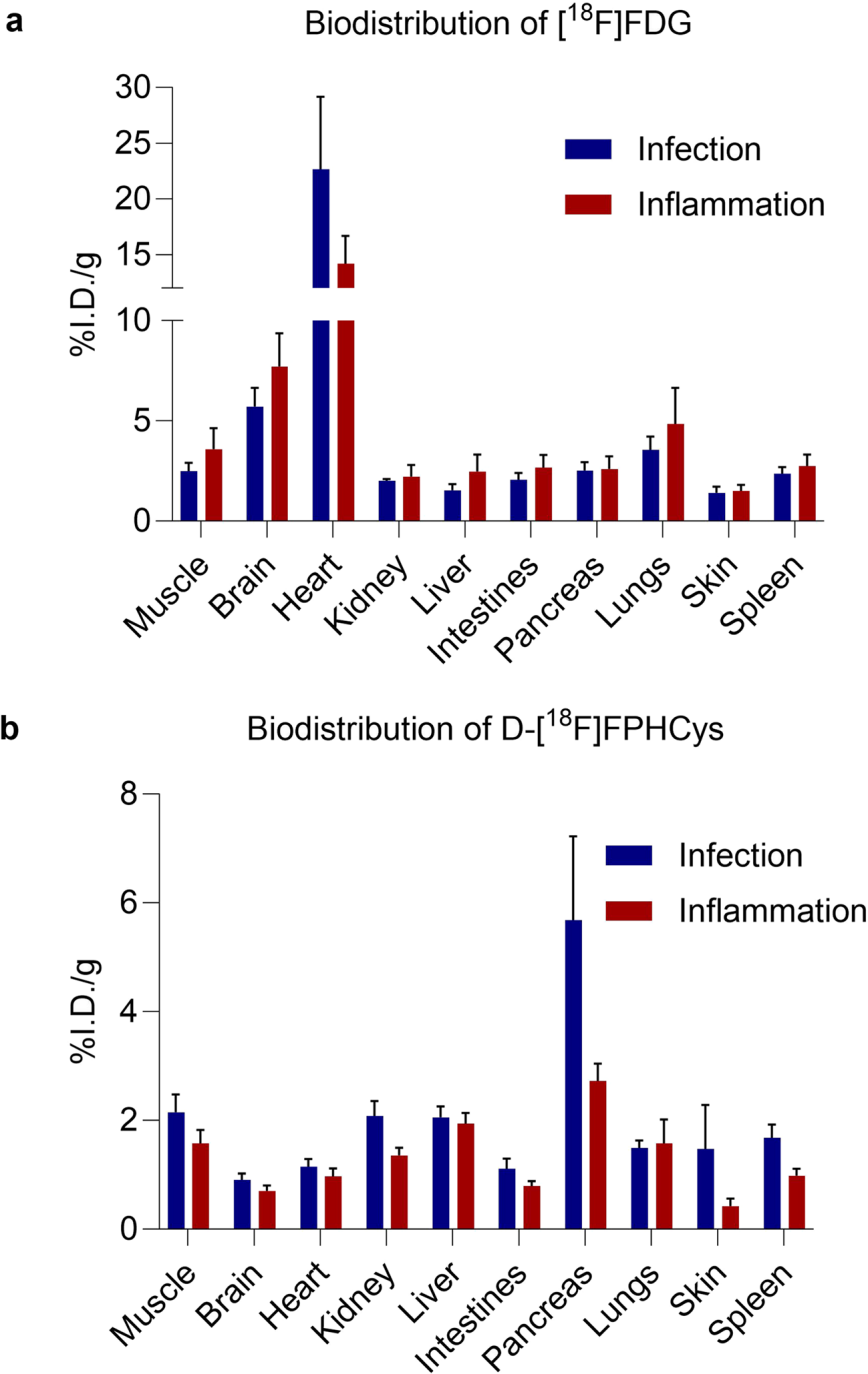
Whole body ex vivo biodistribution in *S. aureus* infection and inflammation models (**a**) [^18^F]FDG (**b**) d-[^18^F]FPHCys.

Whole body *ex vivo* biodistribution of [^18^F]FDG and d-[^18^F]FPHCys in mice with infection and inflammation is shown in Fig. 4a and 4b respectively. The uptake of each radiotracer in the *S. aureus* infection group and the inflammation group is displayed separately. Considering each radiotracer separately, there were no statistically significant differences between the mean uptake in any of the organs in the infection or inflammation groups. The difference in means of pancreatic uptake for d-[^18^F]FPHCys between the two groups was not significant (*p* = 0.21). Full statistical analyses are presented in Tables S1 and S2, in the ESM.

Uptake of d-[^18^F]FPHCys was highest in the pancreas, kidneys and liver, akin to biodistribution data previously reported for this radiotracer in mice for cancer investigations [33]. The prior studies also confirmed good stability of d-[^18^F]FPHCys *in vivo* and that the radioactivity found in the pancreas was > 95% parent compound at 1 h post-injection. The radiotracer was rapidly cleared [33].

### Immunohistochemistry

**Fig. 5:**
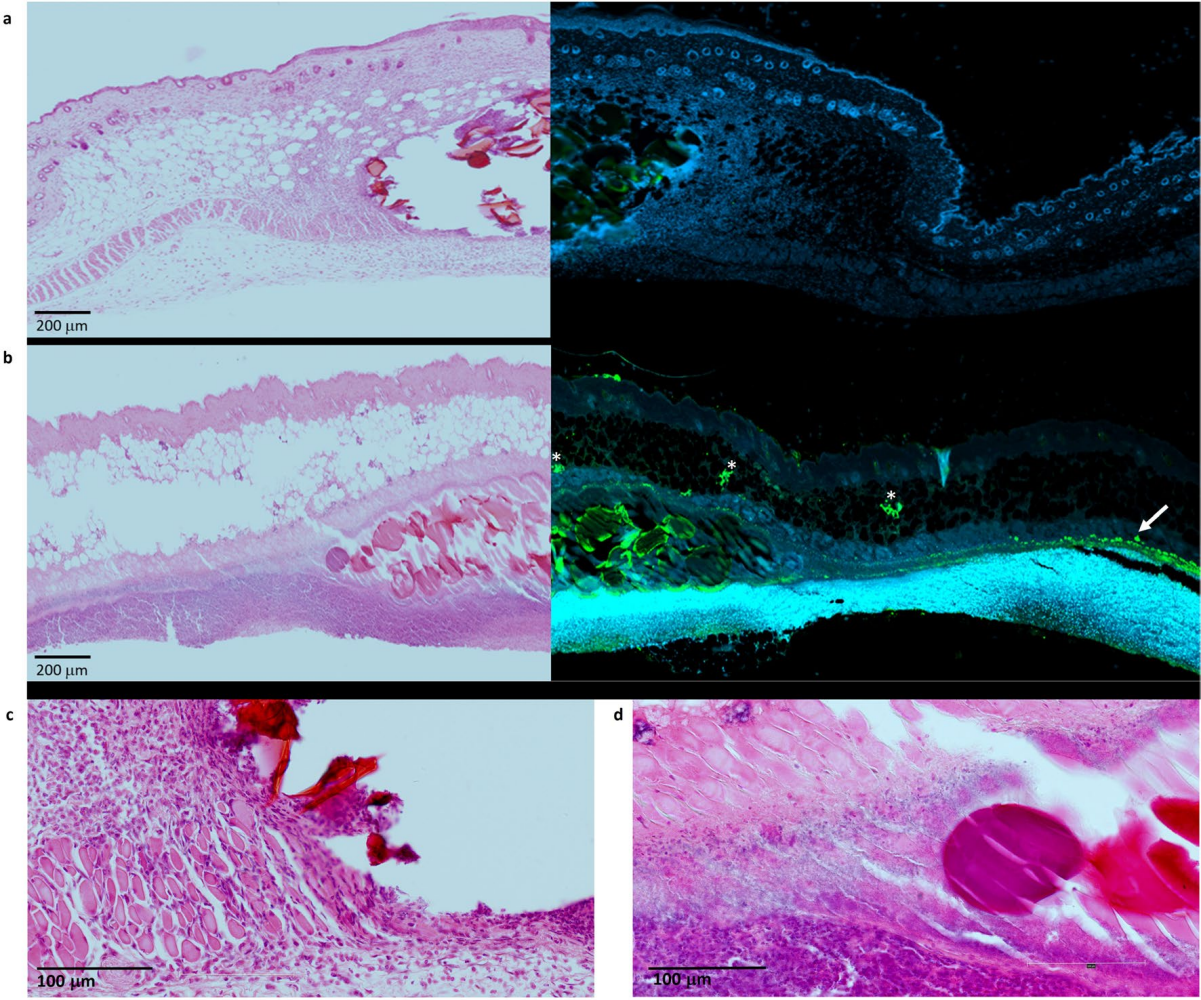
H&E staining of section of mouse skin (left) and visualisation of *S. aureus* by detection with anti-*Staphylococcus* antibody (green, right) for (**a**) sterile inflammation site (**b**) *S. aureus* infection site. Magnified sections showing infiltration of immune cells in (**c**) sterile inflammation with Cytodex bead (**d**) *S. aureus* infection with Cytodex bead. Asterisks indicate clusters of *S. aureus*. Arrow indicates *S. aureus* in muscle layer.

Sections of mouse skin from a sterile inflammation site and an *S. aureus* infection site were stained with hematoxylin and eosin (left panel), and anti-Staphylococcus antibody (right panel) (Fig. 5a and 5b respectively).

The Cytodex beads were surrounded by an infiltration of immune cells in both inflammation and infection. The anti-*Staphylococcus* stain highlights *S. aureus* (green) in the infected section only. The staining pattern indicates clusters of *S. aureus* (asterisks) as well as a lateral development of infection at the basement of the muscle layer (arrow).

## Discussion

The choice of fluorinated d-AAs to investigate was determined by a two factors: (1) feasibility of radiosynthesis from [^18^F]fluoride and (2), likelihood that the d-AA would be incorporated in the bacterial peptidoglycan cell wall, to create the desired contrast between bacteria and human inflammatory cells. ^18^F-Fluorinated AAs can be difficult to access by nucleophilic ^18^F-fluorination radiochemistry [35] and we therefore selected two AA candidates with established synthetic routes. The l-tyrosine (Tyr) derivative l-[^18^F]FET is clinically advanced and is a useful tool for predicting and monitoring treatment response in patients with glioma [36]. The methionine (Met) analogue, [^18^F]FPHCys (as both l- and d-enantiomers) has been evaluated before in cancer imaging [33]. Neither of the d-enantiomers has been evaluated previously for bacterial infection imaging. During the course of this work, d-[^11^C]Met was reported as a promising candidate for bacterial infection imaging, and thus evaluation of its fluorinated analogue d-[^18^F]FPHCys was of interest [27]. d-[^11^C]Alanine (d-[^11^C]Ala) was also described recently, showing good uptake across a panel of bacteria, [29, 37] but although accessible from [^18^F]fluoride, we discounted 3-[^18^F]fluoro-d-Ala because the L-enantiomer rapidly defluorinates *in vivo* [38]. Few other AAs are readily prepared by late-stage radiofluorination.

Exogenous d-AAs are appended to the muropeptides of peptidoglycan by transpeptidase enzymes [39]. Both d-Tyr and d-Met are substrates of these enzymes, which are known to tolerate a variety of unnatural side chains [40–43]. Modification of d-Met and d-Tyr with fluorine-18 was therefore reasonable.

Radiosyntheses of both d-[^18^F]FET and d-[^18^F]FPHCys were achieved as one-pot, two-stage reactions that are amenable to automation, which would be essential for future clinical translation. Furthermore, in both cases, the desired chiral centre was already established in the precursor, and a challenging chiral induction or enantiomer separation were not required in the radiolabelling procedure.

*In vitro* uptake studies confirmed that both d-[^18^F]FET and d-[^18^F]FPHCys became associated with live bacteria but not with heat-killed bacteria, indicating that detection of active infection was feasible. The *in vitro* uptake of d-[^18^F]FPHCys in *S. aureus* was higher than observed by Stewart for d-[^11^C]Met (ca. 20 Bq/10^6^ cells) after 2 h [44], and similar as percentage uptake (ca. 2%) to that observed by Neumann for d-[^14^C]Met, although different *S. aureus* strains were used (Xen29 *vs* ATCC 12600, although Xen29 is a derivative of ATCC 12600) [27, 45, Fig S5 ESM]. Since d-[^18^F]FPHCys is an unnatural analogue of the parent d-Met, we were encouraged that its activity *in vitro* showed at least comparable uptake to the parent under similar assay conditions. Our studies revealed approximately twofold higher-uptake for d-[^18^F]FPHCys than d-[^18^F]FET in both *S. aureus* and *P. aeruginosa*. For this reason, we selected of d-[^18^F]FPHCys for *in vivo* studies.

d-[^18^F]FPHCys was next evaluated *in vivo.* Injection of the Cytodex beads and *S. aureus* for the infection group allowed us to assess a clinically relevant site of co-existing infection and inflammation. *S. aureus* is the most commonly found pathogen in SSI and accounted for around 18% of all SSI in an EU-wide study [8, 46, 47]. In addition to its prevalence in SSI, *S. aureus* is a clinically challenging bacterium which has increasing resistance to antimicrobials, making it a key pathogen for focus in research [48]. The bioluminescent *S. aureus* allowed visualisation of metabolically active *S. aureus* in the animals prior to the biodistribution study. The bioluminescent signal is proportional to the number of metabolically active bacteria present (although factors such as hypoxia and depth effects in imaging prevent accurate calculations of bacterial number *in vivo* from this data). IHC analysis of the infected and inflamed skin sections (after radioactive decay) confirmed leucocyte infiltration in both infection and inflammation sites. By day 4, *S. aureus* infection would have become established and akin to a clinical infection of a foreign body, and is likely to have begun biofilm formation.

In the biodistribution study, d-[^18^F]FPHCys showed increased uptake in *S. aureus* infection site versus inflammation site, which was statistically significant. Although direct comparison of d-[^18^F]FPHCys uptake with d-[^11^C]Met, d-[^11^C]Ala and [^18^F]CF_3_-d-Ala would be informative, this is challenging due to the differing characteristics of the animal models used here and in previously reported studies [27, 29, 31]. The *ex vivo* analysis of radiotracer uptake (as percent injected dose per gram) in the infection site appears generally lower for d-[^18^F]FPHCys than for other reported d-AAs. A comparison of the performance of d-AAs *in vivo* is provided in the ESM (Table S3). A number of reasons may account for this. First, the *S. aureus* infection model used in the present study mimics an established infection, which has been allowed to develop over 4 days. *S. aureus* infections are known to form biofilms [49], potentially creating a barrier to uptake of a blood circulating radiotracer compared with an acute *S. aureus* infection, in which bacteria were injected a few hours before an *in vivo* study. Second, the quantity of bacteria present in the model infection site is an important factor. Typically, acute soft tissue infections are associated with bacterial burden of 10^8^ CFU/mL, but it has been suggested that 10^5^ CFU/mL is a promising threshold for imaging chronic or partially treated infection [7]. Our initial inoculant contained 1 × 10^5^ CFU per infection site, although the burden in the mice at day 4 was not determined. Both these features of our model provide clinically relevant and challenging conditions for a potential radiotracer, as would be faced in a clinical scenario. Future evaluation of d-[^18^F]FPHCys in additional animal models of infection, as well as reducing bacterial load further would be informative.

There was no significant difference in [^18^F]FDG uptake in sites of *S. aureus* infection compared with sterile inflammation in our animal model. This was expected because [^18^F]FDG uptake for infection imaging primarily represents increased glycolytic activity of inflammatory cells, although [^18^F]FDG does accumulate in bacteria [10, 50]. This assertion was borne out in this study by the inability of [^18^F]FDG to distinguish the *S. aureus* infection from inflammation, and supports the conclusion that d-[^18^F]FPHCys is specifically targeting the bacteria. Furthermore, [^18^F]FDG uptake in the inflammation and infection sites in our animal model (ca. 2 %ID/g) was lower than that observed for [^18^F]FDG in the murine myositis model used for evaluation of other d-AA radiotracers (ca. 4 %ID/g) [27, 29, Table S3]. This supports our conclusion that the lower accumulation of d-[^18^F]FPHCys is, at least in part, likely a result of different characteristics of the animal model.

A potential limitation of d-[^18^F]FPHCys, like other radiolabelled d-AAs, is that its uptake requires the bacteria to be in a metabolically active state. For all radiotracers targeting bacterial metabolism, a challenge remains for imaging infection sites that contain populations of quiescent cells, such as during antibiotic treatment. For imaging in these scenarios, radiotracers with uptake that is independent of growth phase may have an advantage. However, drug resistant strains can still be usefully visualised [22, 29].

*In vivo* metabolism of new radiotracers is also a key consideration. d-AAs are possible substrates of d-amino acid oxidase (DAAO), a flavoprotein that catalyses oxidative deamination of neutral d-AAs to form α-keto acids [26]. Although we did not test directly for DAAO metabolism in this study, previous studies in mice showed high metabolic stability of d-[^18^F]FPHCys *in vivo* [33]. One of the advantages of d-CF_3_-Ala is its stability against DAAO, and defluorination (unlike the mono-fluoro derivative, 3-[^18^F]fluoro-d-Ala) [31, 38].

Although *S. aureus* and *P. aeruginosa* are common culprits for SSI, d-[^18^F]FPHCys has not been evaluated across a full panel of pathogens, limiting the scope of this study.

d-[^11^C]Met has recently been evaluated in a first-in-human PET/MR study in healthy volunteers and patients with suspected joint infections [28]. While the results are promising in terms of both a favourable safety profile and an increase of d-[^11^C]Met uptake in suspected infections (although a gold standard for confirmed infection was lacking), an ^18^F-labelled analogue would have practical advantages for future application of d-AA imaging in patients with suspected infection. In our study, d-[^18^F]FPHCys distinguished *S. aureus* infection from sterile inflammation in a clinically relevant mouse model, paving the way for fluorinated d-AA imaging of *S. aureus* infections.

## Conclusions

d-[^18^F]FPHCys is the first reported ^18^F-labelled d-AA able to distinguish *S. aureus* infection from inflammation *in vivo*, although further studies are needed to evaluate its uptake in clinical isolates. d-[^18^F]FPHCys offers practical advantages over d-AA radiotracers reported to date: it has a longer-lived fluorine-18 label versus the ^11^C-labelled d-AAs, and the chiral centre is established in the radiolabelling precursor and retained without racemisation during the radiosynthesis. Direct comparisons of d-[^18^F]FPHCys with d-[^18^F]CF_3_-Ala, which may complementarily detect Gram-positive and Gram-negative bacteria respectively, would be informative for future applications.

### Supplementary Information

Below is the link to the electronic supplementary material.

Supplementary file1 (DOCX 658 KB)

## Data Availability

Chemical synthesis and radiosynthesis data are available in the Electronic Supplementary Material accompanying this manuscript. Additional data is available from the corresponding author on reasonable request.
