# Supplementary material for: Pilot Evaluation of S-(3-[18F]Fluoropropyl)-d-Homocysteine and O-(2-[18F]Fluoroethyl)-d-Tyrosine as Bacteria-Specific Radiotracers for PET Imaging of Infection
Source: Mol Imaging Biol. 2024 Jun 28;26(4):704–13. doi: 10.1007/s11307-024-01929-7 (PMC11282134; doi:10.1007/s11307-024-01929-7)
Supplement: Supplementary file 1 — Supplementary file1 (DOCX 658 KB) [file 11307_2024_1929_MOESM1_ESM.docx]

Electronic Supplementary Material

Evaluation of *S*-(3-[^18^F]fluoropropyl)-d-homocysteine and *O*-(2-[^18^F]fluoroethyl)-d- tyrosine as bacteria-specific radiotracers for PET imaging of infection

Journal: Molecular Imaging and Biology

Helen M. Betts^1,2*^, Jeni C. Luckett^3^, Philip J. Hill^4^

^1^Department of Nuclear Medicine, Nottingham University Hospitals NHS Trust, Queen’s Medical Centre, Nottingham, NG7 2UH, UK. ORCID: 0000-0003-2086-8731.

^2^School of Medicine, University of Nottingham, Queen’s Medical Centre, Nottingham, NG7 2UH, UK.

^3^School of Life Sciences, University of Nottingham, Biodiscovery Institute, University Park, Nottingham, NG7 2RD, UK. ORCID: 0000-0003-3344-7514

^4^ School of Biosciences, University of Nottingham, Sutton Bonington Campus, Sutton Bonington, LE17 5RD, UK. ORCID: 0000-0002-2250-1397

*Corresponding author: Dr Helen M. Betts. Email: [helen.betts@nottingham.ac.uk](mailto:helen.betts@nottingham.ac.uk). Tel: 0044 (0)115 9709172.

Contents

[Synthetic Chemistry 3](#_Toc168650485)

[General 3](#_Toc168650486)

[d-FET 3](#_Toc168650487)

[Precursor to d-[^18^F]FET 5](#_Toc168650488)

[d-FPHCys 7](#_Toc168650489)

[Precursor to d-[^18^F]FPHCys 9](#_Toc168650490)

[Radiochemistry 11](#_Toc168650491)

[HPLC Data 13](#_Toc168650492)

[Radio-HPLC and UV Standards 13](#_Toc168650493)

[Chiral HPLC 14](#_Toc168650494)

[*In vitro* uptake 15](#_Toc168650495)

[Statistical analysis of *ex vivo* data 16](#_Toc168650496)

[d-AA Radiotracers *in vivo* 17](#_Toc168650497)

[References 19](#_Toc168650498)

# Synthetic Chemistry

## General

Chemicals were purchased from Fluorochem or Sigma Aldrich and used as received. d-FET was purchased from ABX, or prepared as described below. Anhydrous solvents were purchased from Sigma Aldrich. NMR was performed using a Bruker Avance 400 MHz spectrometer. MS was performed on a Bruker MicroTOF mass spectrometer using electrospray Ionisation (ESI). Analytical HPLC was performed using an Agilent Series 1200 system with UV detection (λ = 254 nm, unless otherwise noted). The general analytical method used was a gradient system as follows: 0- 1 min 5% B; 1-15 min 5-95% B; 15-18 min 95% B; 18-20 min 95-5% B; 20-25 min 5% B. A flow rate of 1 mL/min was used. Unless otherwise stated, solvent A = H_2_O + TFA (0.1%) and B = MeCN + TFA (0.1%). A Phenomenex Luna C18(2) column (150 mm x 4.6 mm) with guard cartridge was used. Chiral HPLC was performed using a Phenomenex d-penicillamine 3126 column (150 x 4.6 mm) with guard column (30 x 4.6 mm).

All radioactive manipulations were performed in a designated fume hood with lead shielding to reduce operator dose. [^18^F]Fluoride was purchased from The University of Hull’s Positron Emission Tomography Research Centre (PETRC) and was supplied as [^18^F]fluoride in H_2_[^18^O]O. Sep-pak QMA-carb cartridges were purchased from Waters.

## d-FET

**Methyl-(*R*)-2-((*tert*-butoxycarbonyl)amino)-3-(4-(2-fluoroethoxy)phenyl)propanoate, Boc-d-Tyr(EtF)OMe**

According to the literature method^[[1]](#endnote-1)^, Boc-d-Tyr-OMe (1.01 g, 3.4 mmol, 1 eq.) was dissolved in DMF (6 mL) and K_2_CO_3_ (3.23 g, 23.4 mmol, 6.9 eq.) was added. 18-Crown-6 (0.10 g, 0.4 mmol, 0.1 eq.) and 2-fluoroethyl tosylate (0.82 g, 3.8 mmol, 1.1 eq.) were added and the reaction stirred at room temperature for 70 h. H_2_O (25 mL) was added to the reaction, and the product extracted into EtOAc (2 x 30 mL). The combined organics were washed with brine (2 x 30 mL), dried (MgSO_4_), and evaporated under reduced pressure. The product was purified by silica chromatography (4:1 hexane:EtOAc) yielding a colourless oil, which solidified on standing. (0.30 g, 26.0%). **^1^H NMR** (CDCl_3_) δ 7.07 (d, *J* = 8.6 Hz, 2H), 6.88 (d, *J* = 8.6 Hz, 2H), 4.97 (d, *J* = 7.9 Hz, 1H), 4.77 (dt, *J* = 47.4, 4.2 Hz, 2H), 4.57 (m, 1H), 4.22 (dt, *J* = 27.7, 4.2 Hz, 2H), 3.73 (s, 3H), 3.09-2.96 (m, 2H), 1.44 (s, 9H). **MS** [M + Na]^+^ *m/z* 364.1542 (calc for C_17_H_24_FNNaO_5_^+^ 364.1531). **HPLC** *R_t_* = 13.3 min.

**(*R*)-2-[(tert-butoxycarbonyl)amino]-3-[4-(2-fluoroethoxy)phenyl]propanoic acid, Boc-d-Tyr(EtF)-OH**

Following the procedure for the L-enantiomer^1^, Boc-d-Tyr(EtF)-OMe (0.14 g, 0.4 mmol) was dissolved in THF (4 mL), cooled in an ice bath, and LiOH.H_2_O (aq. 2 M, 1 mL) was added. The reaction was stirred and allowed to warm to room temperature over 2 h, and then the THF was removed under vacuum. The solution was acidified with KHSO_4_ (5% aq., 20 mL), and the product was extracted into EtOAc (2 x 25 mL). The combined organic layers were dried over MgSO_4_, and solvent evaporated, yielding the product as a colourless oil. (0.13 g, 98.7%). **NMR** (CDCl_3_) δ 7.11 (d, *J* = 8.6 Hz, 2H), 6.87 (d, *J* = 8.6 Hz, 2H), 4.92 (d, *J* = 7.3 Hz, 1H), 4.74 (dt, *J* = 47.4, 4.2 Hz, 2H), 4.57 (m, 1H), 4.19 (dt, *J* = 27.8, 4.2 Hz, 1H), 3.17-3.00 (m, 2H), 1.42 (s, 9H). **HPLC** *R_t_* = 11.8 min.

**(*R*)-2-amino-3-[4-(2-fluoroethoxy)phenyl]propanoic acid trifluoroacetate salt, (d-FET)**

Boc-d-Tyr(EtF)-OH (25.6 mg, 0.08 mmol) was dissolved in CH_2_Cl_2_ (300 µL) and TFA (150 µL) was added. The reaction stirred at room temperature for 40 min, after which the volatiles were removed. Diethyl ether (5 mL) was added, which precipitated a white solid that was filtered off and rinsed with diethyl ether (2 x 5 mL). The product was dried *in vacuo*. (24.4 mg, 96.2%). Product identity was confirmed by comparison of HPLC with the commercially available material, and by MS. **MS** [M + H]^+^ *m/z* 228.1025 (calc for C_11_H_15_FNO_3_^+^ 228.1030); [M + Na]^+^ *m/z* 250.0838 (calc for C_11_H_14_FNNaO_3_^+^ 250.0850). **HPLC** *R_t_* = 7.0 min.

## Precursor to d-[^18^F]FET

***Tert*-butyl trityl-D-tyrosinate, Trt-d-Tyr-O*^t^*Bu**

H-d-Tyr-O*^t^*Bu (1.19 g, 5.0 mmol, 1 eq.) was dissolved in THF (20 mL) and cooled in an ice bath. Triethylamine (697 µL, 0.51 g, 5.0 mmol, 1 eq.) was added, followed by trityl chloride (1.40 g, 5.0 mmol, 1 eq.). The ice bath was removed, and the reaction was allowed to warm to room temperature. The reaction stirred for 48 h, after which it was diluted with water (40 mL) and extracted with EtOAc (2 x 25 mL). The combined organic layers were washed with brine (3 x 20 mL), dried over MgSO_4_ and evaporated to dryness. The product was purified by column chromatography on silica (hexane:EtOAc 4:1). The product was isolated as colourless crystalline solid (2.17 g, 90.4%). **^1^H NMR** (CDCl_3_) δ 7.49 (d, 6H, *J* = 7.4 Hz), 7.30-7.22 (m, 6H), 7.22-7.15 (m, 3H), 7.09 (d, 2H, *J* = 8.0 Hz), 6.74 (d, 2H, *J* = 8.0 Hz), 5.16 (br s, 1H), 3.50 (dd, 1H, *J* = 13.6 Hz, 7.0 Hz), 2.89-2.73 (m, 2H), 2.62 (1H, d, *J* = 9.0 Hz), 1.11 (s, 9H). The NMR data was in agreement with the literature reference for the L-enantiomer.^[[2]](#endnote-2)^ **MS** [M + Na]^+^ *m/z* 502.2357 (calc for C_32_H_33_NNaO_3_^+^ 502.2353). **HPLC** A variant of the general gradient method was used: 0- 1 min 50% B; 1-15 min 50-95% B; 15-18 min 95% B; 18-20 min 95-50% B; 20-25 min 50% B. Solvent A = MeCN, solvent B = H_2_O). *R_t_* = 15.6 min.

***tert*-butyl 3-[4-(2-bromoethoxy)phenyl]-2-(tritylamino)propanoate, Trt-d-Tyr(EtBr)-O*^t^*Bu**

Trt-d-Tyr-O*^t^*Bu (0.50 g, 1.0 mmol) was dissolved in dibromoethane (8 mL) and 18-crown-6 (0.028 g, 0.1 mmol) and K_2_CO_3_ (0.98 g, 7.1 mmol) were added. The reaction was heated at 80 °C for 70 h. The reaction was cooled and H_2_O (40 mL) added. The product was extracted into EtOAc (3 x 30 mL) and washed with brine (3 x 30 mL). The organic layers were dried over MgSO_4_ and the solvent removed *in vacuo*. The crude product was purified by column chromatography on silica (9:1 hexane:EtOAc). The product was isolated as a colourless oil (0.58 g, 95.3%). **^1^H NMR** (CDCl_3_) δ 7.46-7.43 (m, 6H), 7.25-7.19 (m, 6H), 7.17-7.12 (m, 5H), 6.86-6.82 (m, 2H), 4.28 (t, 2H, *J* = 6.3 Hz), 3.63 (t, 2H, *J* = 6.3 Hz), 3.51-3.44 (m, 1H), 2.87-2.73 (m, 2H), 2.58 (d, 1H, *J* = 9.6 Hz), 1.08 (s, 9H).

**MS** [M + Na]^+^ *m/z* 608.1750 (calc for C_34_H_36_BrNNaO_3_^+^ 608.1771). **HPLC** A variant of the general gradient method was used: 0- 1 min 50% B; 1-15 min 50-95% B; 15-18 min 95% B; 18-20 min 95-50% B; 20-25 min 50% B. Solvent A = MeCN, solvent B = H_2_O). *R_t_* = 18.9 min.

***Tert*-butyl 3-{4-[2-(tosyloxy)ethoxy]phenyl}-2-(tritylamino)propanoate,** **Trt-d-Tyr(EtOTs)-O*^t^*Bu**

Trt-d-Tyr(EtBr)-O*^t^*Bu (0.30 g, 0.5 mmol) was dissolved in MeCN (8 mL) and silver tosylate (0.30 g, 1.0 mmol) was added. The reaction stirred at reflux for 24 h, after which the reaction was cooled and the solid filtered off. The THF was evaporated, then the residue redissolved in EtOAc. The mixture was washed with H_2_O (2 x 30 mL) and brine (2 x 30 mL), dried over MgSO_4_ and evaporated. The crude product was purified by silica column chromatography, first eluting with 9:1 hexane:EtOAc (no product elution) then 4:1 hexane:EtOAc. The product was isolated as a glassy solid (0.11 g, 30.8%). **^1^H NMR** (CDCl_3_) δ 7.82 (d, 2H, *J* = 8.3 Hz), 7.45-7.42 (m, 6H), 7.33 (d, 2H, *J* = 8.0 Hz), 7.24-7.18 (m, 6H), 7.17-7.08 (overlapping m, 5H), 6.73-6.6.69 (m, 2H), 4.38-4.34 (m, 2H), 4.15-4.11 (m, 2H), 3.49-3.42 (m, 1H), 2.85-2.71 (m, 2H), 2.56 (br d, 1 H, *J* = 8.7 Hz), 2.43 (s, 3H), 1.07 (s, 9H). **MS** [M + H]^+^ *m/z* 678.2878 (calc for C_41_H_44_NO_6_S^+^ 678.2884); [M + Na]^+^ *m/z* 700.2685 (calc for C_41_H_43_NNaO_6_S^+^ 700.2703). **HPLC**: A variant on the general gradient method was used: 0- 1 min 70% B; 1-15 min 70-95% B; 15-18 min 95% B; 18-20 min 95-70% B; 20-25 min 70% B. Flow rate of 1 mL/min. Solvent A = MeCN, solvent B = H_2_O). *R_t_* = 16.3 min.

## d-FPHCys

**(*2R,2'R*)-4,4'-Disulfanediyl*bis*{2-[(*tert*-butoxycarbonyl)amino]butanoic acid}**

Using a similar procedure to Zhu *et al*,^[[3]](#endnote-3)^ D-homocystine (2.29 g, 8.5 mmol) was partially dissolved in sodium carbonate (10 % aq., 90 mL) and dioxane (50 mL) and cooled in an ice bath. Di-*tert*-butyl dicarbonate (3.51 g, 16.1 mmol, 2 eq.) was added as a solution in dioxane (5 mL) and the reaction was allowed to warm to room temperature. The reaction stirred for 22.5 h, after which a white precipitate was observed. The reaction was adjusted to pH 4 with citric acid (aq. 10%), and extracted into EtOAc (3 x 75 mL). The EtOAc layers were washed with brine (2 x 100 mL), dried over MgSO_4_, and the solvent evaporated under reduced pressure yielding a white solid (3.19g, 79.8%). **^1^H NMR** (DMSO-d_6_) δ 12.56 (s, 1H), 7.15 (d, 1H, *J* = 8.1 Hz), 3.99 (dt, 1H, *J* = 4.5 Hz, *J* = 9.1 Hz), 2.72, (t, 2H), 2.09-1.99 (m, 1H), 1.95-1.84 (m, 1H), 1.38 (s, 9H). The NMR data was in agreement with the literature report.^[[4]](#endnote-4)^

**Di-*tert*-butyl 4,4'-disulfanediyl(*2R,2'R*)-*bis*{2-[(tert-butoxycarbonyl)amino]butanoate}**

(*2R,2'R*)-4,4'-disulfanediyl*bis*{2-[(*tert*-butoxycarbonyl)amino]butanoic acid} (1.54 g, 3.3 mmol) was dissolved in dichloromethane (20 mL), and *t*-butyl-2,2,2-trichloroacetimidate (TBTA) (3.50 g, 16.0 mmol, 5 eq.) was added as a solution in dichloromethane (5 mL). The reaction stirred at room temperature for 24 h, after which time a fine white solid was observed. The solid was filtered off, and filtrate evaporated under reduced pressure. The crude product was purified by column chromatography on silica (9:1 hexane:EtOAc). The product was isolated as a waxy white solid (1.27 g, 66.5%). **^1^H NMR** (CDCl_3_) δ 5.10 (br d, 1H, *J* = 7.7 Hz), 4.30-4.21 (br m, 1H), 2.76-2.57 (m, 2H), 2.25-2.15 (m, 1H), 2.03-1.97 (m, 1H), 1.47 (s, 9H), 1.44 (s, 9H). The NMR spectrum was in agreement with the literature values.^3^

***Tert*-butyl (*tert*-butoxycarbonyl)-D-homocysteinate, Boc-d-HCys-O*^t^*Bu**

Following the procedure of Wnuk^[[5]](#endnote-5)^, di-*tert*-butyl 4,4'-disulfanediyl(*2R,2'R*)-*bis*{2-[(tert-butoxycarbonyl)amino]butanoate} (1.01 g, 1.7 mmol) was dissolved in degassed DMF (5 mL), then *tris*(2-carboxyethyl) phosphine hydrochloride (0.55 g, 1.9 mmol, 1.1 eq.) was dissolved in H_2_O (1.2 mL) and added to the reaction in 3 portions. The reaction was stirred under N_2_ at room temperature for 24 h. NaHCO_3_ (sat. aq.) was added and the product was extracted in EtOAc (3 x 25 mL), then washed with brine (3 x 30 mL). The organic layer was dried over MgSO_4_ and solvent evaporated under reduced pressure, leaving the product as a colourless oil in high purity (by NMR) for direct use in the next step (1.0 g, 99%). After removing samples for characterisation, the product used immediately in the next steps. **^1^H NMR** (CDCl_3_) δ 5.06 (d, 1H, *J* = 7.1 Hz), 4.34-4.27 (br m, 1H), 2.64- 2.49 (m, 2H), 2.13-2.03 (m, 1H), 1.94-1.84 (m, 1H), 1.47 (s, 9H), 1.44 (s, 9H). The NMR spectrum was in agreement with the literature reported values.^3^ **MS** [M + H]^+^ *m/z* 292.1574 (calc for C_13_H_26_NO_4_S^+^ 292.1577); [M + Na]^+^ *m/z* 314.1403 (calc for C_13_H_25_NNaO_4_S^+^ 314.1397).

***S*-(3-fluoropropyl)-D-homocysteine hydrochloride, d-[^19^F]FPHCys.HCl, via *tert*-butyl *N*-(*tert*-butoxycarbonyl)-*S*-(3-fluoropropyl)-d-homocysteinate**, **Boc-d-FPHCys-O*^t^*Bu**

Boc-d-HCys-O*^t^*Bu (0.50 g, 1.7 mmol) was dissolved in anhydrous, degassed DMF (3 mL) and K_2_CO_3_ (0.47 g, 3.4 mmol 2 eq.) was added, followed by 3-fluoropropyl tosylate (0.60 g, 2.6 mmol, 1.5 eq.). The reaction headspace was purged with nitrogen, and the reaction stirred at room temperature for 5 days. H_2_O (30 mL) was added, and the product extracted into EtOAc (3 x 25 mL) and washed with brine (3 x 30 mL). The organic extracts were dried over MgSO_4_ and solvent removed under vacuum. (0.49 g) The product was partially purified by column chromatography on silica (hexane:EtOAc 9:1), but it was difficult to achieve complete separation of FPrOTs from the product. **^1^H NMR** of the mixture showed approximate ratio of 7:3 FPrOTs:Boc-d-FPHCys-O*^t^*Bu, by comparison of the integrals of the C*H_2_*F peaks. **MS** confirmed both species present. Boc-D-FPHCys-O*^t^*Bu [M + H]^+^ *m/z* 352.1951 (calc for C_16_H_31_FNO_4_S^+^ 352.1952); [M + Na]^+^ *m/z* 374.1767 (calc for C_16_H_30_FNNaO_4_S^+^ 374.1772); [M + NH_4_]^+^ *m/z* 369.2195 (calc for C_16_H_34_FN_2_O_4_S^+^ 369.2218). Also present [FPrOH + Na]^+^ *m/z* 255.0467 (calc for C_10_H_13_FNaO_3_S^+^ 225.0462). **HPLC** Boc-d-FPHCys-O*^t^*Bu (λ = 220 nm) *R_t_* = 13.0 min. FPrOTs (λ = 254 nm) *R_t_* = 13.1 min. The mixture was used directly in the next step, at which point the fluoropropyl tosylate was easily separated. The mixture of Boc-d-FPHCys-O*^t^*Bu and FPrOTs (0.13g) was dissolved in THF (0.34 mL) and HCl (6 M, 0.70 mL) was added. The reaction stirred at room temperature for 3 h, and then the volatiles removed under vacuum. The resulting residue was thoroughly washed with EtOAc (3 x 10 mL), which removed the residual FPrOTs, leaving a white semi-crystalline solid (11.9 mg, 45 % based on proportion of Boc-d-FPHCys-O^t^Bu present from NMR analysis of the starting mixture). **^1^H NMR** (D_2_O) δ 4.62 (dt, 2H, *J* = 5.7 Hz, *J* = 47.0 Hz), 4.16 (t, 1H, *J* = 6.3 Hz), 2.77-2.70 (m, 4H), 2.36-2.15 (m, 2H), 2.09-1.94 (m, 2H). The NMR spectrum was in agreement with the literature report.^6^ **MS** [M + H]^+^ *m/z* 196.0803 (calc for C_7_H_15_FNO_2_S^+^ 196.0802); [M + Na]^+^ *m/z* 218.0621 (calc for C_7_H_14_FNNaO_2_S^+^ 218.0621). **HPLC** (λ = 220 nm) *R_t_* = 6.4 min. **HPLC** analysis was also performed at λ =254 nm, confirming the absence of FPrOTs at *R_t_* = 13.1 min.

## Precursor to d-[^18^F]FPHCys

***Tert*-butyl *N*-(*tert*-butoxycarbonyl)-*S*-(3-hydroxypropyl)-D-homocysteinate, Boc-d-HCys(PrOH)-O*^t^*Bu**

Following the procedure of Bourdier,^[[6]](#endnote-6)^ Boc-d-HCys-O*^t^*Bu (0.50 g, 1.7 mmol) was dissolved in anhydrous DMF (3 mL) and degassed. K_2_CO_3_ (0.47 g, 3.4 mmol, 2 eq.) was added the reaction headspace purged with N_2_. 3-Bromopropanol (0.23 mL, 2.58 mmol, 1.5 eq.) was added and the reaction stirred overnight at room temperature under N_2_. Water was added and the product was extracted with EtOAc (3 x 20 mL), then washed with brine (3 x 30 mL). The organic layer was dried over MgSO_4_ and solvent evaporated under vacuum. The product was purified by column chromatography on silica (hexane:EtOAc 4:1), yielding a colourless oil (0.41 g, 67.6%). **^1^H NMR** (CDCl_3_) δ 5.12 (d, 1H, *J* = 6.1 Hz), 4.34-4.26 (m, 1H), 3.81-3.71 (m, 2H), 2.65 (t, 2H, *J* = 7.0 Hz), 2.59-2.54 (m, 2H), 2.13-2.03 (m, 1H), 1.94-1.78 (m, 4H), 1.47 (s, 9H), 1.44 (s, 9H). The NMR data was in agreement with the literature values.^6^ **MS** [M + Na]^+^ *m/z* 372.1809 (calc for C_16_H_31_NNaO_5_S^+^ 372.1815). **HPLC** (λ = 220 nm) *R_t_* = 13.0 min.

***Tert*-butyl *N*-(*tert*-butoxycarbonyl)-*S*-[3-(tosyloxy)propyl]-D-homocysteinate, Boc-HCys(PrOTs)O*^t^*Bu**

Following the procedure of Bourdier^6^, Boc-d-HCys(PrOH)-O*^t^*Bu (0.33 g, 1.0 mmol) was dissolved in MeCN (7.5 mL) in a two-necked round bottom flask. *N,N,N’,N’*-Tetramethyl-1,6-hexane diamine (406 µL, 1.9 mmol, 2 eq.) was added and the reaction headspace purged with N_2_. Tosyl chloride (0.27 g, 1.4 mmol, 1.5 eq.) was added, and a white precipitate was observed. The reaction stirred at room temperature for 3 h. The reaction mixture was diluted with H_2_O (25 mL), extracted with EtOAc (3 x 25 mL) and the organic extracts washed with brine (2 x 30 mL). The EtOAc was evaporated under reduced pressure. The crude product was purified by column chromatography on silica (4:1 hexane:EtOAc), yielding a glassy solid (0.16 g, 32.9%). **^1^H NMR** (CDCl_3_) δ 7.84-7.74 (m, 2H), 7.35 (d, 2H, *J* = 8.0 Hz), 5.07 (br d, 1H, *J* = 7.4 Hz), 4.27-4.19 (br m, 1H), 4.13 (t, 2H, *J* = 6.1 Hz), 2.53 (t, 2H, *J* = 7.1 Hz), 2.50-2.43 (m, 2H), 2.45 (s, 3H), 2.10-1.96 (br m, 1H), 1.90 (qu, 2H, *J* = 6.6 Hz), 1.87-1.77 (m, 1H), 1.46 (s, 9H), 1.44 (s, 9H). NMR data was in agreement with literature reported values.^6^ **MS** [M + Na]^+^ *m/z* 526.1909 (calc for C_23_H_37_NNaO_7_S_2_^+^ 526.1904); [M + H]^+^ *m/z* 504.2084 (calc for C_23_H_38_NO_7_S_2_^+^ 504.2084); [M + NH_4_]^+^ *m/z* 521.2345 (calc for C_23_H_41_N_2_O_7_S_2_^+^ 521.2350). **HPLC** A variation on the general method was used. 0- 1 min 50% B; 1-15 min 50-95% B; 15-18 min 95% B; 18-20 min 95-50% B; 20-25 min 50% B. A = H_2_O, B = MeCN. *R_t_* = 12.8 min.

# Radiochemistry

Radiolabelling was performed manually inside a fume cupboard equipped with lead shielding.

*D-[^18^F]FET*

[^18^F]Fluoride in [^18^O]H_2_O (~ 450 MBq) was loaded to a QMA carbonate cartridge (Waters). The cartridge was eluted with a mixture of Kryptofix-222 (5 mg), potassium carbonate (50 µL, 0.1 M, aq), H_2_O (40 µL) and acetonitrile (800 µL) into a v-vial equipped with a stirrer vane. The vial was heated at 110 °C under air flow to dry the [^18^F]fluoride, without stirring. Two further portions of anhydrous acetonitrile (2 x 0.5 mL) were added and similarly dried. The precursor **3** (6 mg) was dissolved in anhydrous acetonitrile (0.5 mL) and added to the dried [^18^F]fluoride. The reaction was heated (with stirring) to 100 °C for 10 min, after which the acetonitrile was evaporated under a stream of air at 100 °C for 1 min. HCl (0.3 mL, 2 M) was added to the vessel for the deprotection reaction, which stirred for 5 min at 100 °C. The reaction was allowed to cool, then NaOH (0.3 mL, 2 M) was added, and the pH of the solution checked and further adjusted with HCl (2 M) or NaOH (2 M) until pH 7. The product was purified using HPLC equipped with a Phenomenex Synergi Hydro-RP column (250 x 10 mm) 4µ 80A, eluting with a pre-mixed solution of EtOH (10%) in NaH_2_PO_4_ (0.01 M, aq.) at a flow rate of 2 mL/min and UV detection at 254 nm. *R_t_* = 16.7 min. Only a portion of the reaction mixture was purified to minimise radiation dose to the operator. The overall activity yield was estimated using a multiplication factor to account for the entire reaction volume. The mean estimated activity yield of isolated product was 22.0% in a reaction time of 88 min. The mean molar activity at end of synthesis was 2.3 GBq/µmol.

Analytical HPLC was performed using a Phenomenex Luna C18(2) column, 150 x 4.6 mm. Radio- and UV-detectors were arranged in series (λ = 254 nm). A gradient method was used in which solvent A = H_2_O + 0.1% TFA, and B = MeCN + 0.1% TFA. 0-1 min 5%B; 1-15 min 5-95%B; 15-18 min 95%B; 18-20 min 95-5%B; 20-25 min 5%B. The flow rate was 1.0 mL/ min. *R_t_* = 7.1 min. Radiochemical purity >99%.

Chiral analysis was performed using a Phenomenex Chirex 3126 d-penicillamine column (150 x 4.6 mm) with guard column (30 x 4.6 mm), with eluent 86% [Cu(II)SO_4_] (2 mM aq) and 14% ^i^PrOH, with flow rate of 1.0 mL/min, and detection at 254 nm. *R_t_* = 21 min. Enantiomeric purity >99%.

*D-[^18^F]FPHCys*

d-[^18^F]FPHCys was prepared using a similar method to Bourdier.^6^ [^18^F]Fluoride (~600 MBq) in [^18^O]H_2_O was diluted with H_2_O (to a total volume of approx. 1 mL), and loaded to a QMA carbonate extraction cartridge (Waters). The cartridge was eluted with a mixture of Kryptofix-222 (10 mg), potassium oxalate (2.6 mg), potassium carbonate (50 µg), H_2_O (200 µL) and acetonitrile (800 µL), directly into a v-vial equipped with a stirrer bar. The [^18^F]fluoride was dried under a stream of air at 110 °C. Two further portions of acetonitrile (2 x 0.5 mL) were added then dried. The precursor (5 mg) was dissolved in anhydrous acetonitrile (0.5 mL) and heated with the dried [^18^F]fluoride for 10 min at 100 °C. The acetonitrile was evaporated for 1 min, and HCl (2 M, 0.3 mL) was added to remove the protecting groups. The deprotection reaction was heated to 100 °C for 5 min. After allowing to cool, the mixture was neutralised with NaOH (aq. 2 M, ca. 0.3 mL) and passed through an alumina-N cartridge, to remove unreacted [^18^F]fluoride. The resulting solution was purified by HPLC using the same column and flow rate as d-[^18^F]FET, but using eluent containing EtOH (5%) in NaH_2_PO_4_ (0.01 M, aq.). UV detection was set at 220 nm. *R_t_* = 15.4 min. The mean activity yield of isolated product was 10.3 % in a synthesis time of 80 min. The molar activity at end of synthesis was at least 1.7 GBq/µmol. Exact values were not calculated because the quantity of d-[^19^F]FPHCys present in formulation was below the UV detection limit of the HPLC equipment. Minimum molar activity was calculated using the instrument detection limit (λ = 220 nm) of 1 µg/mL.

Analytical HPLC was performed as for d-[^18^F]FET, but UV detection was set to 220 nm. *R_t_* = 6.4 min. Radiochemical purity >99%. Chiral HPLC analysis was performed as for d-[^18^F]FET, with eluent 89% [Cu(II)SO_4_] (2 mM aq.) and 11% ^i^PrOH, flow rate 0.9 mL/min, and detection at 254 nm. *R_t_* = 17 min (d-enantiomer). Enantiomeric purity >99%.

# HPLC Data

## Radio-HPLC and UV Standards

**D-FET**


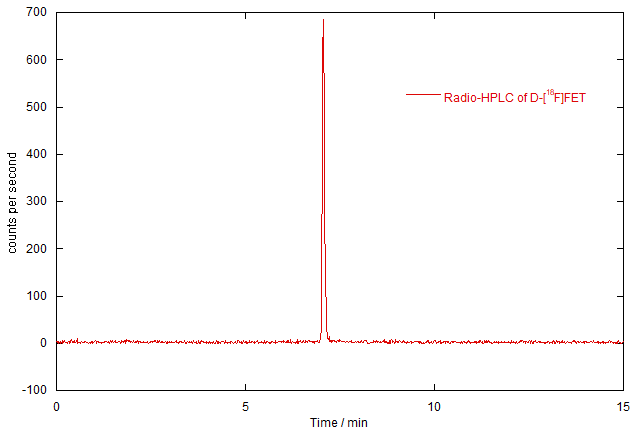

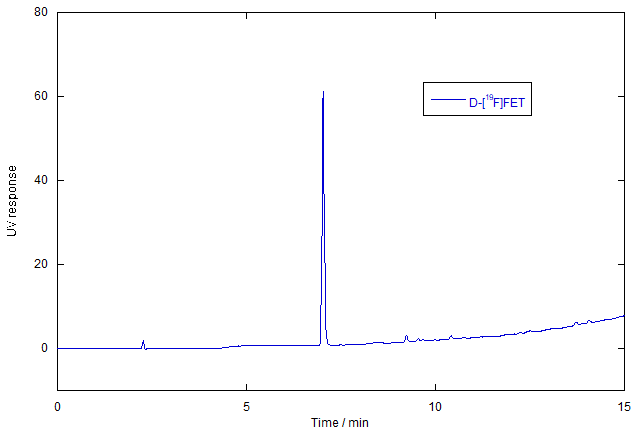


Figure S1: Analytical HPLC traces of d-FET. a. Radio-HPLC of formulated d-[^18^F]FET. b. UV-HPLC of non-radioactive reference material. *R_t_* d-FET= 7.1 min.

**D-FPHCys**


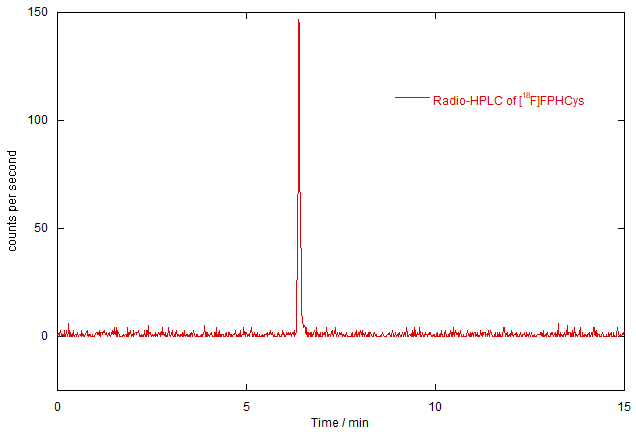

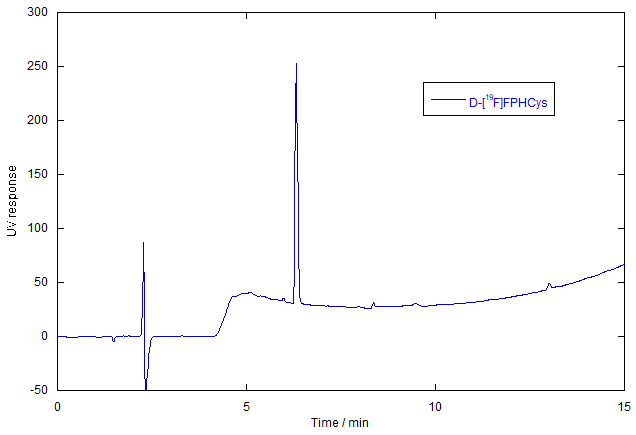


Figure S2: Analytical HPLC traces of d-FPHCys. a. Radio-HPLC of formulated d-[^18^F]FPHCys. b. UV-HPLC of non-radioactive reference material. *R_t_* d-FPHCys =6.4 min.

## Chiral HPLC

**d-FET**

a
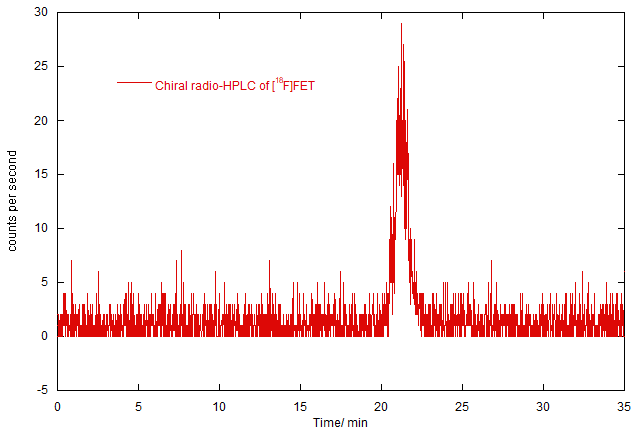
b
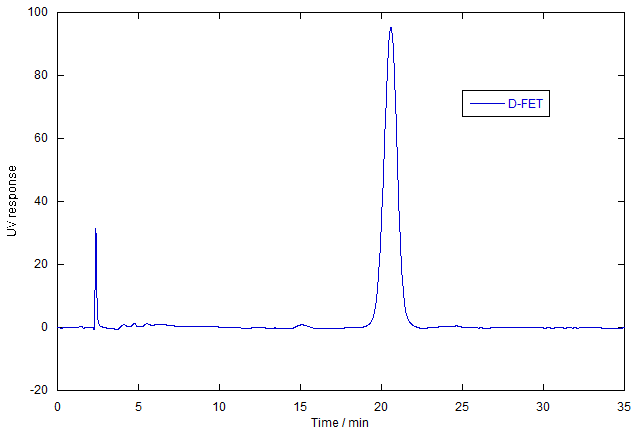


Figure S3: Chiral HPLC of d-FET. a. Radio-HPLC trace of formulated d-[^18^F]FET. b. UV-HPLC trace of non-radioactive reference. *R_t_* d-FET = 21 min.

**d-FPHCys**

a
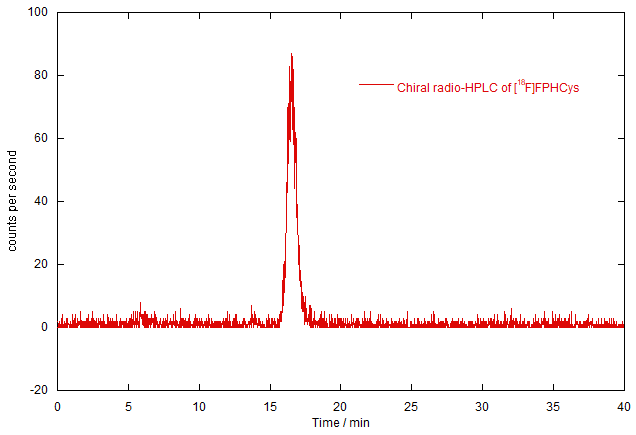
b
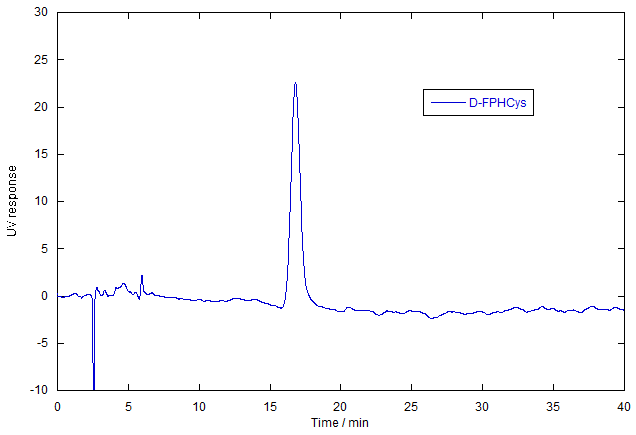


Figure S4: Chiral HPLC of d-FPHCys. a. Radio-HPLC trace of formulated d-[^18^F]FPHCys. b. UV-HPLC trace of non-radioactive reference. *R_t_* d-FPHCys = 17 min.

# *In vitro* uptake


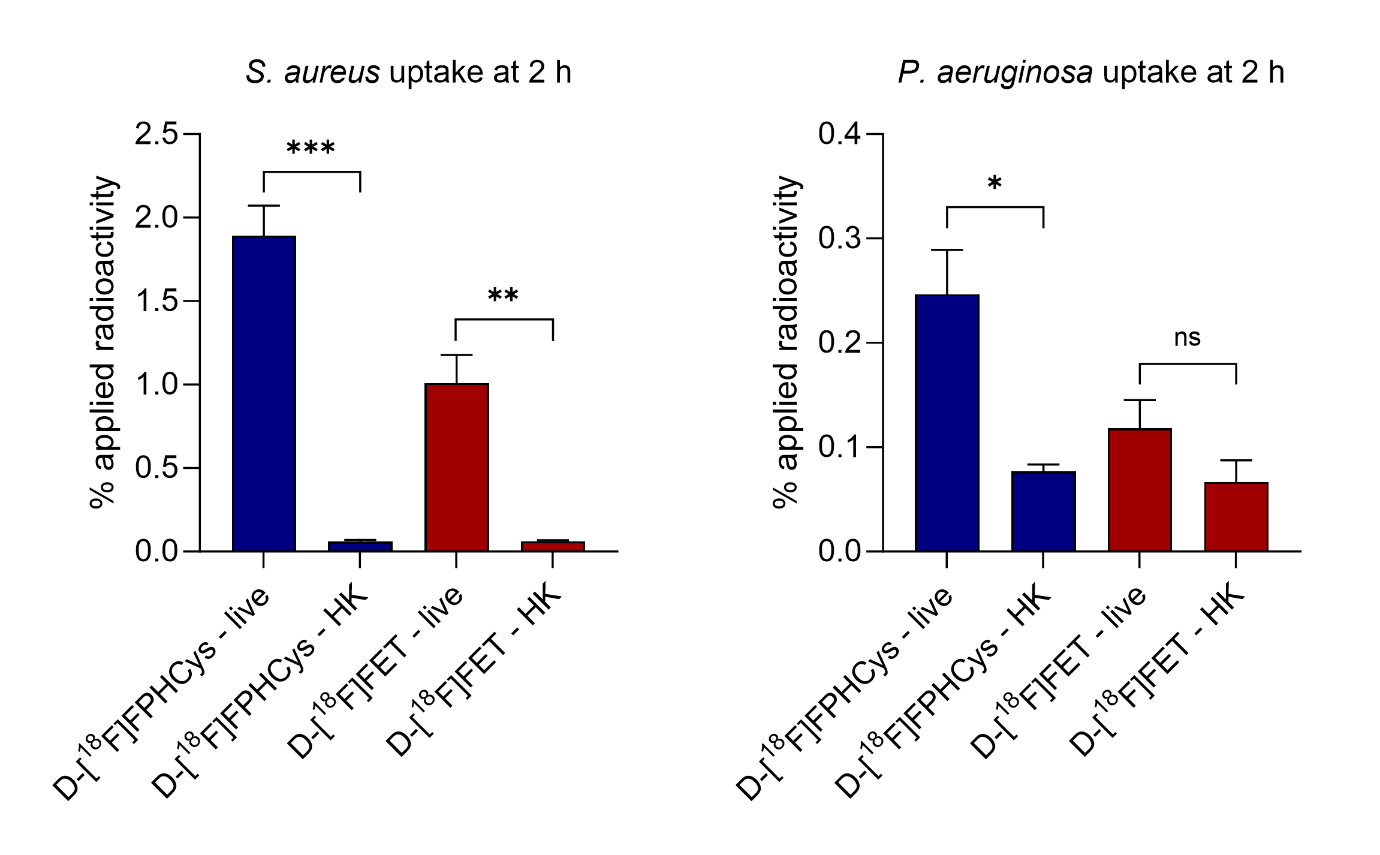


Figure S5: *In vitro* uptake of d-[^18^F]FPHCys and d-[^18^F]FET in *S. aureus* and *P.* *aeruginosa*. Data presented as percentage applied activity associated with the bacteria after 2h incubation. HK = heat-killed.

# Statistical analysis of *ex vivo* data

Table S1: Data from *ex vivo* biodistribution study of d-[^18^F]FPHCys. Data shows mean ± SEM.

| Organ | Mean (%I.D./g)  Infection | Mean (%I.D./g)  Inflammation | *p* value (t-test) |
| --- | --- | --- | --- |
| Cytodex bead site | 0.91 ±0.05 | 0.64 ±0.07 | 0.016 |
| Muscle | 2.15 ±0.33 | 1.57 ±0.25 | 0.28 |
| Brain | 0.91 ±0.12 | 0.71 ±0.09 | 0.31 |
| Heart | 1.15 ±0.14 | 0.97 ±0.15 | 0.43 |
| Kidney | 2.07 ±0.28 | 1.36 ±0.14 | 0.12 |
| Liver | 2.06 ±0.20 | 1.93 ±0.20 | 0.71 |
| Intestines | 1.11 ±0.19 | 0.79 ±0.09 | 0.30 |
| Pancreas | 5.68 ±1.54 | 2.72 ±0.32 | 0.22 |
| Lungs | 1.49 ±0.14 | 1.58 ±0.44 | 0.82 |
| Skin | 1.48 ±0.81 | 0.42 ±0.14 | 0.39 |
| Spleen | 1.68 ±0.25 | 0.98 ±0.12 | 0.088 |

**[^18^F]FDG**

Table S2: Data from *ex vivo* biodistribution study of [^18^F]FDG. Data shows mean ± SEM.

| Organ | Mean (%I.D./g)  Infection | Mean (%I.D./g)  Inflammation | *p* value (t-test) |
| --- | --- | --- | --- |
| Cytodex bead site | 2.04 ±0.83 | 2.08 ±1.50 | 0.94 |
| Muscle | 2.49 ±0.42 | 3.58 ±1.05 | 0.36 |
| Brain | 5.71 ±0.94 | 7.72 ±1.65 | 0.32 |
| Heart | 22.7 ±6.50 | 14.2 ±2.48 | 0.25 |
| Kidney | 2.01 ±0.09 | 2.23 ±0.58 | 0.72 |
| Liver | 1.53 ±0.32 | 2.48 ±0.83 | 0.31 |
| Intestines | 2.12 ±0.38 | 2.67 ±0.63 | 0.47 |
| Pancreas | 2.52 ±0.43 | 2.59 ±0.64 | 0.93 |
| Lungs | 3.55 ±0.66 | 4.85 ±1.79 | 0.51 |
| Skin | 1.42 ±0.30 | 1.51 ±0.29 | 0.83 |
| Spleen | 2.37 ±0.33 | 2.74 ±0.57 | 0.58 |

# d-AA Radiotracers *in vivo*

Table S3: Summary of d-AA radiotracer performance *in vivo,* and [^18^F]FDG comparisons where available.

|  | **Radiotracer** | **Animal model**  **(mouse)** | **CFU administered** | **Time for infection to develop prior to *in vivo* study** | **Infection uptake** | **Inflammation uptake** | **Notes** | **Reference** |
| --- | --- | --- | --- | --- | --- | --- | --- | --- |
| 1 | d-[^18^F]FPHCys | Foreign body - Cytodex beads  *S. aureus* | 1 x 10^5^ | 4 d | 0.91 %ID/g | 0.64 %ID/g | 60 min | *This work* |
| 2 | [^18^F]FDG | Foreign body - Cytodex beads  *S. aureus* | 1 x 10^5^ | 4 d | 2.0 %ID/g | 2.1 %ID/g | 60 min | *This work* |
| 3 | d-[^11^C]Met | Murine myositis  *S. aureus*  *E. coli* | CFU not stated | 8 h | 1.0 %ID/cc^†‖^  1.5 %ID/cc^†^  3.5 %ID/g^‡^  0.8 %ID/cc^†‖^  1.4 %ID/cc^†^  1.7 %ID/g^‡^ | 0.05 %ID/cc^† ‖^  0.6 %ID/cc^†^  0.5 %ID/g^‡^  0.1 %ID/cc^†‖^  0.7 %ID/cc^†^  0.3 %ID/g^‡^ | Inflammation 10 x HK bacteria  PET 60-70 min  *Ex vivo* 70 min | Neumann 2017^7^ |
| 4 | [^18^F]FDG | Murine myositis  *S. aureus* | CFU not stated | 8 h | 4.5 %ID/g^†^  3.4 %ID/g^‡^ | 7 %ID/g^†^  3.9 %ID/g^‡^ | 45-60 min | Neumann 2017^[[7]](#endnote-7)^ |
| 5 | d-[^11^C]Ala | Murine myositis  *S. aureus*  *E. coli*  *Pneumonia: P. aeruginosa*  Vertebral discitis-osteomyelitis*:*  *S. aureus (rat)* | 5 x 10^6^  5 x 10^6^  2 x 10^6^  5 x 10^6^ | 10 h  10 h  6 h  4 d | 8 % ID/g^‡^  5 %ID/g^‡^  1.5 %ID/cc^†^  8% ID/g^‡^  2 %ID/cc^†^ | 2 % ID/g^‡^  1.5 %ID/g^‡^ | Inflammation 10 x HK bacteria  PET 60-85 min  *Ex vivo* 95 min  0.8 %ID/cc^†^  Background region  4 %ID/g^‡^  Background - (non-infected lung treated with vehicle)  0.6 %ID/cc^†^ Background region | Parker 2020^[[8]](#endnote-8)^ |
| 6 | [^18^F]FDG | Murine myositis  *S. aureus*  *E. coli* | 5 x 10^6^  5 x 10^6^ | 10 h  10 h | 4 %ID/g^‡^  4 %ID/g^‡^ | 4 %ID/g^‡^  4 %ID/g^‡^ | PET 60-85 min  *Ex vivo* 95 min | Parker 2020^8^ |
| 7 | d-[^11^C]Gln | Murine myositis dual model  *Multidrug resistant S. aureus*  *E. coli* | 10^6^-10^7^ | 12 h  12 h | 0.15 (SUV)^†^  0.12 (SUV)^†^ | 0.06 (SUV)^†^  Turpentine inflammation  0.4 (SUV)^†^ | PET 20-40 min  Inflammation HK bacteria | Renick 2021^[[9]](#endnote-9)^ |
| 8 | [^18^F]FDG | Inflammation - turpentine | - | 3-4 d | - | 5.0 (SUV)^†^ | PET 60-70 min Turpentine inflammation | Renick 2021^9^ |
| 9 | d-[^18^F]CF_3_-Ala | Murine myositis  *E. coli* | 2 x 10^7^ | 12 h | 5.5 %ID/cc^†^  1.1% ID/g^‡^ | 2.0 %ID/cc^†^  0.7 %ID/g^‡^ | Inflammation 10x HK bacteria  PET 0-90 min  *Ex vivo* 100 min | Sorlin 2024^[[10]](#endnote-10)^ |

All data shown is best estimation of numerical data from displayed graphs. ^†^Obtained from PET imaging. ^‖^Normalised to muscle. ^‡^Obtained from *ex vivo* dissection. HK = Heat killed.

# References

1. Betts HM, Milicevic Sephton S, Tong C *et al*. Synthesis, in vitro evaluation, and radiolabeling of fluorinated puromycin analogues: potential candidates for PET imaging of protein synthesis. J Med. Chem. 2016:59;9422-9430. [↑](#endnote-ref-1)
2. Fan M, Zhou W, Jiang Y, Ma D. CuI/Oxalamide catalysed couplings of (hetero)aryl chlorides and phenols for diaryl ether formation. Angew Chem Int Ed. 2016;55:6211-6215. [↑](#endnote-ref-2)
3. Zhu J, Hu X, Dizin S, Pei D. Catalytic mechanism of S-ribosylhomocysteinase (LuxS):  direct observation of ketone intermediates by ^13^C NMR spectroscopy. J Am Chem Soc. 2003;125:13379-13381. [↑](#endnote-ref-3)
4. Serafinowski P, Dorland E, Harrap KR. Synthesis and antiviral activity of some new *S*-adenosyl-l-homocysteine derivatives. J Med Chem. 1992;35:4576-4583. [↑](#endnote-ref-4)
5. Wnuk SF, Robert J, Sobczak AJ, Meyers BP *et al*. Inhibition of *S*-ribosylhomocysteinase (LuxS) by substrate analogues modified at the ribosyl C-3 position. Bioorg Med Chem. 2009;17:6699-6706. [↑](#endnote-ref-5)
6. Bourdier T, Shepherd R, Berghofer P *et al*. Radiosynthesis and biological evaluation of L- and D-*S*-(3-[^18^F]fluoropropyl)homocysteine for tumor imaging using positron emission tomography. J Med Chem. 2011;54:1860-1870. [↑](#endnote-ref-6)
7. Neumann KD, Villanueva-Meyer JE, Mutch CA *et al*. Imaging active infection in vivo using d-amino acid derived PET radiotracers. Sci Rep. 2017;7:7903. [↑](#endnote-ref-7)
8. Parker MFL, Luu JM, Schulte B *et al*. Sensing living bacteria in vivo using alanine-derived ^11^C radiotracers. ACS Central Sci 2020;6:155-165. [↑](#endnote-ref-8)
9. Renick PJ, Mulgaonkar A, Co CM *et al*. Imaging of actively proliferating bacterial infections by targeting the bacterial metabolic footprint with d-[5-^11^C]-glutamine. ACS Infect Dis. 2021;7:347-361. [↑](#endnote-ref-9)
10. Sorlin AM, López-Alvarez M, Biboy J *et al*. Peptidoglycan-targeted [^18^F]3,3,3-trifluoro-d-alanine tracer for imaging bacterial infection. JACS Au 2024;4:1039-1047. [↑](#endnote-ref-10)
